# High-throughput virtual search of small molecules for controlling the mechanical stability of human CD4

**DOI:** 10.1016/j.jbc.2024.107133

**Published:** 2024-03-02

**Authors:** Antonio Reifs, Alba Fernandez-Calvo, Borja Alonso-Lerma, Jörg Schönfelder, David Franco, Mariano Ortega-Muñoz, Salvador Casares, Concepcion Jimenez-Lopez, Laura Saa, Aitziber L. Cortajarena, David De Sancho, Eider San Sebastian, Raul Perez-Jimenez

**Affiliations:** 1 Center for Cooperative Research in Biosciences (CIC bioGUNE), Basque Research and Technology Alliance (BRTA), Derio-Bizkaia, Spain; 2 Center for Cooperative Research in Nanoscience (CIC nanoGUNE), Basque Research and Technology Alliance (BRTA), Donostia-San Sabestian, Spain; 3 Glaxosmithkline, Rixensart, Belgium; 4 Faculty of Science, Department of Organic Chemistry, University of Granada, Granada, Spain; 5 Faculty of Science, Department of Physical Chemistry, University of Granada, Granada, Spain; 6 Department of Microbiology, University of Granada, Granada, Spain; 7 Center for Cooperative Research in Biomaterials (CIC biomaGUNE), Basque Research and Technology Alliance (BRTA), Donostia-San Sebastian, Spain; 8 Ikerbasque, Basque Foundation for Science, Bilbao, Spain; 9 Donostia International Physics Center (DIPC), San Sebastian, Spain; 10 Faculty of Chemistry, Applied Chemistry Department, University of the Basque Country (UPV/EHU), San Sebastian, Spain

**Keywords:** cluster of differentiation 4 (CD4), protein mechanics, small molecule, protein–drug interaction, molecular docking

## Abstract

Protein mechanical stability determines the function of a myriad of proteins, especially proteins from the extracellular matrix. Failure to maintain protein mechanical stability may result in diseases and disorders such as cancer, cardiomyopathies, or muscular dystrophy. Thus, developing mutation-free approaches to enhance and control the mechanical stability of proteins using pharmacology-based methods may have important implications in drug development and discovery. Here, we present the first approach that employs computational high-throughput virtual screening and molecular docking to search for small molecules in chemical libraries that function as mechano-regulators of the stability of human cluster of differentiation 4, receptor of HIV-1. Using single-molecule force spectroscopy, we prove that these small molecules can increase the mechanical stability of CD4D1D2 domains over 4-fold in addition to modifying the mechanical unfolding pathways. Our experiments demonstrate that chemical libraries are a source of mechanoactive molecules and that drug discovery approaches provide the foundation of a new type of molecular function, that is, mechano-regulation, paving the way toward mechanopharmacology.

Numerous proteins in the cell withstand mechanical loads while performing their function (1, 2, 3, 4). This is especially significant for cell-surface proteins located in the extracellular matrix, which are essential for the communication between cells in the extracellular milieu (5, 6, 7). Reacting to mechanical force through conformational changes is crucial for these cell-surface proteins, translating a physical signal into an intracellular signaling process (8, 9, 10), or establishing physical connection with other cells (11). Over 1400 cell-surface proteins compose the human surfaceome, including integrins, intercellular adhesion molecules, and cluster of differentiation (CD) molecules (12), which highlights the importance of protein mechanics in the cell. Similarly, viruses and bacteria use their own surface proteins to establish anchoring with cell-surface molecules to initiate infection (13). Again, the mechanical stability of these protein–protein interactions plays a crucial role in the success of the infection process (14, 15), implying an important function of mechanical force in viral entry and bacterial adhesion (16, 17, 18). In fact, it is known that perturbating such interaction may result in avoidance of infection (19).

In the past years, efforts have been made toward designing protocols to control the mechanostability of proteins. For instance, an elegant work by Rivas *et al.* demonstrated that blocking the formation of isopeptide bonds in *Streptococcus pyogenes* pilus proteins, it is possible to interfere with the pili formation (20). This interference could potentially alter the adhesion capabilities of the bacterium. Also, it is well known that mutations in strategic locations show effectiveness in altering the mechanical stability of proteins (21, 22, 23, 24); nevertheless, mutations are irreversible and most often go in the destabilizing direction. Other studies have demonstrated that antibody binding or metal chelation can also alter the mechanical stability of proteins (16, 25). Altogether, these studies have provided a wealth of information regarding protein mechanics in biological systems; however, introducing mutations, using antibodies or metal ions may have some complications for practical implementation as mechano-modulators.

Here, we propose a mutation-free approach to alter protein mechano-stability utilizing small molecules. Our technology combines high-throughput virtual screening (HTVS) of compound libraries, molecular docking, and single-molecule atomic force spectroscopy (smAFS). HTVS allows searching thousands of compounds from virtual chemical libraries similar to procedures commonly utilized in drug discovery (26, 27, 28). The molecular docking allows targeting specific regions previously known as potential relevant mechanical sites. We apply this approach to CD4 protein, a coreceptor present in T lymphocytes membrane, which is involved on antigen recognition, but also, it is the primary receptor of HIV-1. We have identified three small molecules in smAFS experiments, which probe their ability to modify and enhance CD4 mechanical stability, thus acting as protein mechanical stability regulators (PROMESRs). We propose these PROMESR molecules as a proof of concept of mechano-active molecules discovered by means of a drug discovery pharmacology-based approach, bringing the possibility of a new class of mechano-drugs. We propose that PROMESR might be useful molecules not only to alter the mechanical stability of cell-surface protein but also that of any protein whose function relies on its mechanical integrity. Thus, PROMESR may be useful to interfere with any protein–protein interaction process that occurs with the intervention of forces, such as those happening between microbes and host cells or cell–cell interaction.

## Results

### HTVS of a compound library

Our initial step focused on the search of small molecules capable of binding regions of CD4 that can potentially influence the mechanical stability of the molecule. We designed a virtual screening search of commercially available small molecules available in the ZINC chemical library (https://zinc.docking.org/), using Glide (https://newsite.schrodinger.com/platform/products/glide/) software from Schrödinger Suite (29), which docks molecules in the structure of CD4 (PDB ID: 1WIP). We first performed the validation of the docking protocol as described in the Supplementary material (Figs. S1–S3). We restricted the search and docking to domains D1 and D2 of CD4 by creating three partially overlapping grids that cover the whole structure (Fig. S4). The first grid focused on D1, another one D2, and the third one focusing on the interface of D1D2. This strategy derives from our previous knowledge on the mechanical stability of CD4 domains (16), from which we know that the continuous β-strand shared by domains D1 and D2 and the interface between these domains play a crucial role in the mechanical integrity of the tandem (16). In fact, it was demonstrated that an antibody named Ibalizumab (commercialized as Trogarzo), which precisely binds the D1D2 interface (30), has a strong mechanical effect on the stability of CD4 D1D2 (16). Thus, the interface between the domains is a clear target in our search.

**Figure 1:**
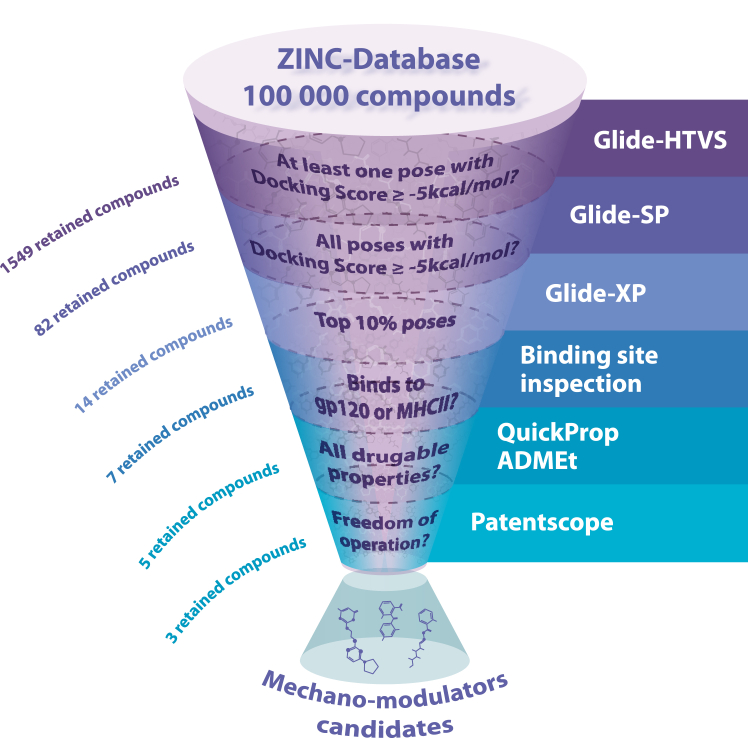
**Workflow used to identify protein mechano-modulators.** The key properties of an ideal mechano-modulator were established as follows: (1) it should display a strong binding to the problem protein. To achieve this, we make use of Glide-HTVS, Glide-SP, and Glide-XP; (2) it should not interfere with the protein-binding site or any relevant epitope/active site, depending on the problem protein; (3) it should have optimal ADMEt properties; and should enjoy of a complete freedom of operation at the industrial property level. ADME, absorption, distribution, metabolism, and excretion; HTVS, high-throughput virtual screening; SP, standard precision; XP, extra precision.

**Figure 2:**
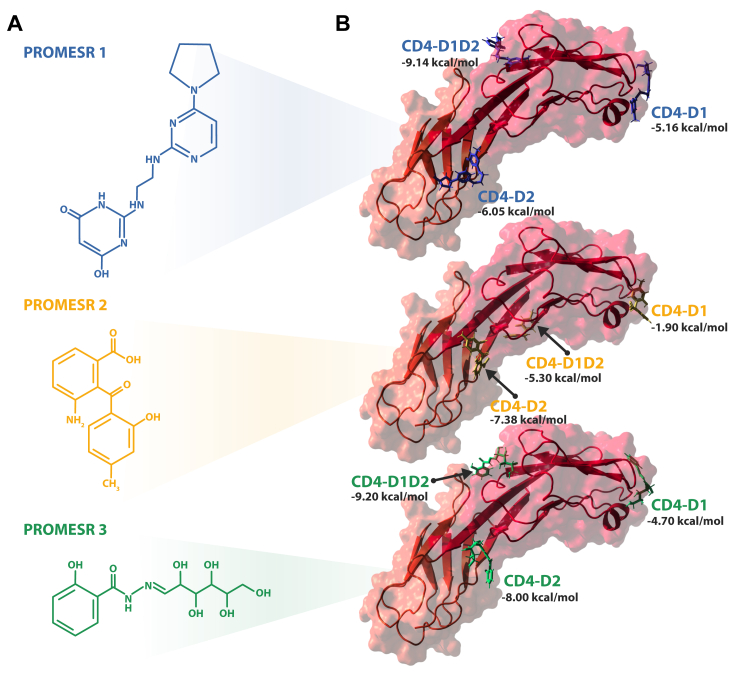
**Structural predicted interactions of PROMESR and CD4 domains.***A*, chemical structure of PROMESR 1 (ZINC65466948), PROMESR 2 (ZINC0048160), and PROMESR 3 (ZINC05514670). *B*, surface and *cartoon representation* of CD4 domains 1 and 2. In *stick representation* appear all the poses derived from the XP docking procedure of (*top*) PROMESR 1, (*middle*) PROMESR 2, and (*bottom*) PROMESR 3. CD, cluster of differentiation; PROMESR, protein mechanical stability regulator; XP, extra precision.

We then followed a multistep approach to retain molecules with at least one with ligand binding energy (docking score) below a given threshold of −5 kcal/mol. Values below −5 kcal/mol are considered strong binding, and approved drugs display values in the range −5 to −10 kcal/mol (31). This resulted in 1549 compounds with binding energies below that threshold established in the HTVS phase. Figure 1 depicts a schematic representation of the search protocol, which is presented in more detail in Fig. S5 and described in the Experimental procedures section. Subsequent redocking of the selected compounds using the standard precision level of Glide (standard precision mode), which performs a harder torsional refinement and sampling of the conformations, promoted the number of compounds of interest to be narrowed down to 82. The latter displayed a binding energy with values below −5 kcal/mol for at least one of the three grids under study. A final extra precision (XP level of Glide (32), assigning a XP Gscore) docking procedure of the compounds selected so far, to penalize ligands that do not fit well to the receptor conformation, filtered out all but 14 compounds. Subsequent binding site analysis excluded ligand/poses interfering with forbidden binding sites (FBS). FBS were defined in the CD4 structure; residues 35 to 52 and 55 to 60 were defined as major histocompatibility complex (MHC) class II binding epitope (Fig. S6) (ref (33)), retaining eight compounds. A QuickProp ADMEt analysis retained five compounds of which three enjoy freedom of operation. In summary, three were the compounds from the ZINC lead-like subset of compounds that fulfilled all the filtering criteria established in the present study. These potential mechanical regulators of CD4 (ZINC65466948, ZINC00481608, and ZINC05514670 in ZINC database) will be referred to as PROMESR 1, 2 and 3 (Fig. 2), respectively. The structures of the PROMESR are shown in Figure 2*A*. PROMESR were obtained either by commercial providers or by chemical synthesis.

### Binding and interaction of PROMESR with CD4D1D2

**Figure 3:**
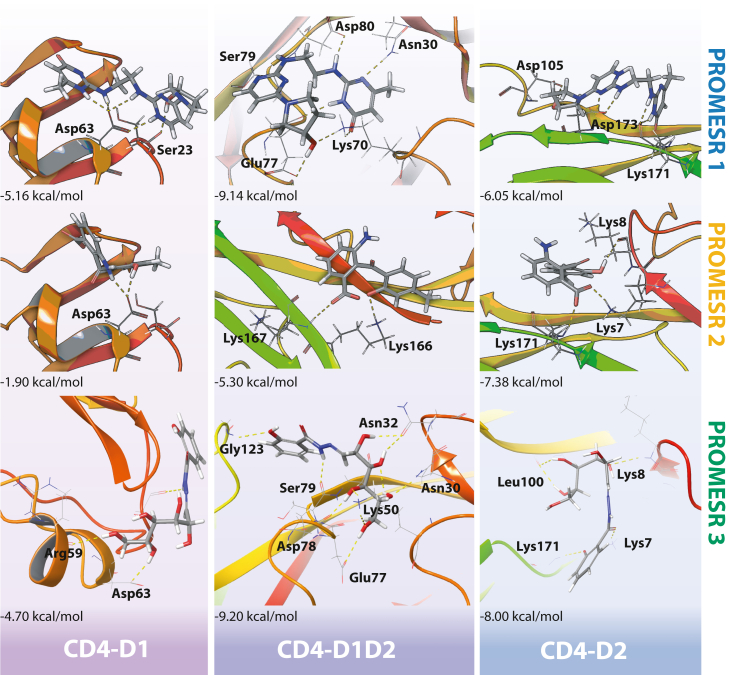
**Detailed predicted interactions of PROMESR and residues in CD4 domains.** PROMESR 1 (*top*), PROMESR 2 (*middle*), and PROMESR 3 (*bottom*) are the most efficient binding modes to domain 1 tip (*left*), domains 1 and 2 interface (*middle*), and domain 2 (*right*). XP Gscore values (kcal/mol) are indicated in *bold* below each pose. H-bonds established between each molecule and key residues in CD4 domains are indicated with *dashed lines*. CD, cluster of differentiation; PROMESR, protein mechanical stability regulator; XP, extra precision.

An analysis of the binding energetics, binding sites, and binding modes of PROMESR to CD4 revealed that these compounds can bind to distinct regions of the receptor with different predicted affinities (Fig. 2*B*); however, some of these regions are similar for the three small molecules. PROMESR 1 may preferentially bind to the interface between domain 1 and domain 2, with an XP Gscore of −9.14 kcal/mol, but can also bind D1 and D2 with an XP Gscore of −5.16 and −6.05 kcal/mol, respectively. PROMESR 2 may preferentially bind to domain 2, with an XP Gscore of −7.38 kcal/mol. It also binds close to the interface between D1 and D2 with an XP Gscore value −5.30 kcal/mol but on the opposite side that of PROMESR 1. Finally, PROMESR 3 binds the interface between domain 1 and 2 in the back side of the tandem with docking score value of −9.20 kcal/mol but can also bind D2 with an XP Gscore value of −8.0 kcal/mol and weakly to D1. In addition, as observed in Figure 3, PROMESR establish key interactions with residues in CD4, mostly charged and polar residues indicating the electrostatic nature of the interactions. A detailed interaction diagram is shown in Fig. S7 for each PROMESR molecule. Interestingly, the interaction of PROMESR 3 near the interface between D1 and D2 involves residues Ser79 and Glu77 in D1, which have been also shown to be important in the interaction of Trogarzo and CD4 (30). Nevertheless, given the considerable smaller size of the PROMESR with respect to Trogarzo, we do not expect many interacting residues to be common between both molecules. Moreover, we were surprised to see that some of the PROMESR poses bind very similar locations in CD4 domains, for example, PROMESR 1 and 3 in the three grids, or poses in D1 and D2, which highlight these regions as potential druggable sites (Fig. S8).

For informative purposes on the translational potential, we have run a prediction of multiple physically significant descriptors and pharmaceutically relevant properties such as absorption, distribution, metabolism, and excretion (ADME) descriptors of single conformers of PROMESR using QikProp software (https://newsite.schrodinger.com/platform/products/qikprop/). The ADME descriptors are shown in Table S1 and were compared with those of 95% of known drugs. As observed, PROMESR 1 and 2 have optimal properties with respect to their drugability. In this line, an analysis of the Lipinskis rule of five (34) (if # stars = 0 fulfills all the rules) implies that PROMESR 1 and 2 have properties like 95% of those drugs found in the market, which suggest that the search process provides small-molecules that are even potential drugs. For instance, human oral absorption value equal to 3 implies that these two drugs may likely be good candidates to be orally administered in *in vivo* preclinical and clinical tests. PROMESR 3, with six H-bond donors and # stars = 1 is still an excellent oral candidate. We have also carried out a standard test of the cytotoxicity on HEK293 cells of the different PROMESR and compared it with that of Trogarzo at different concentrations. Very similar levels of cell viability were obtained with all PROMESR and Trogarzo (Fig. S9).

### smAFS of PROMESR molecules

**Figure 4:**
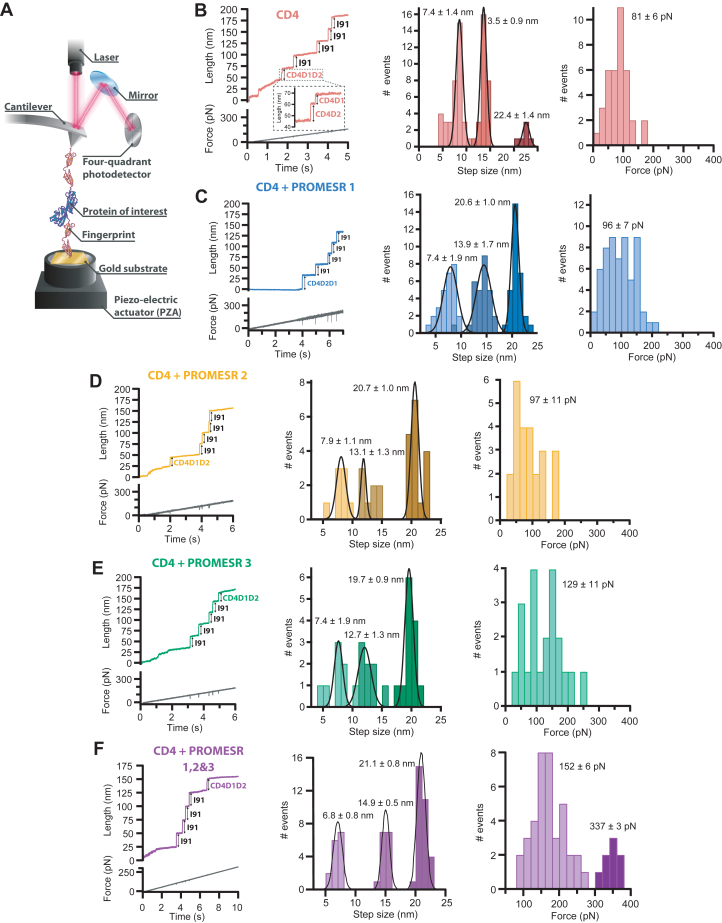
**smAFS experiments of PROMESR and CD4 in force-ramp mode.***A*, representation of the (I91)_2_-CD4D1D2-(I91)_2_ polyprotein construct on an smAFS setup. The I91 modules are used as molecular fingerprint. The polyprotein is attached to a gold substrate in one end and a cantilever in the other end. *B*, typical force-ramp trace of (I91)_2_-CD4D1D2-(I91)_2_ polyprotein and histograms of step size and initial unfolding force (n = 37). *C*, trace and histograms in the presence of PROMESR 1 (n = 56), (*D*) **PROMESR** 2 (n = 29), (*E*) **PROMESR** 3 (n = 29), and (*F*) the three PROMESR together (n = 46). Different colors have been used to identify each combination. Step size is reported as mean ± SD. Force is reported as mean ± SEM. CD, cluster of differentiation; PROMESR, protein mechanical stability regulator; smAFS, single-molecule atomic force spectroscopy.

To test the mechanical effect of PROMESR effectors on CD4 domains, we used smAFS. We first designed a polyprotein composed of CD4, domains D1 and D2, flanked by handles of two-domains I91 subunits from human cardiac titin, resulting in the polyprotein (I91)2CD4D1D2(I91)2 to which we apply a calibrated mechanical force (Fig. 4*A*). We have successfully used this construct before to prove the effect of force on CD4 (16).

The four I91 subunits are used as a mechanical fingerprint, due to the well-known properties of this subdomain in smAFS systems and have been used to study the mechanics of many other proteins (10, 14). For the smAFS experiments, we chose the so-called force-ramp mode, in which the force applied to the polyprotein is ramped up at a constant speed of 33 pN·s-1. Force-ramp experimental data is characterized by a typical ramped staircase, in which each step represents the unfolding process of one subdomain from the polyprotein construct (Fig. 4*B*). In the case of the polyprotein used here, we identify four equal steps from I91 domains. We have measured an initial unfolding force of 128 ± 5 pN (mean ± SEM) for I91 domains. This initial unfolding force represents the average minimum force at which I91 domains start unfolding. For I91 domains, we determine step size of 24.9 ± 2.6 nm (mean ± SD), which is in agreement with the size expected at the loading rate that we applied (16) (Fig. S10).

We also identify one or two additional steps corresponding to our protein of interest CD4D1D2. In the case of CD4D1D2 alone, we mostly observe two unfolding steps, although we also observed the unfolding of the tandem in a single step, which means that both domains are being unfolded simultaneously. We determine an average initial unfolding force of 81 ± 6 pN. In the case of two-step unfolding of the tandem, we measure step size of 7.4 ± 1.4 nm and 13.5 ± 0.9 nm for D1 and D2, respectively (Fig. 4*B*). In the case of the one step unfolding, the step size observed is 22.4 ± 1.4 nm, which is the sum of the two domains. These values are consistent with those reported before by us (16).

**Figure 5:**
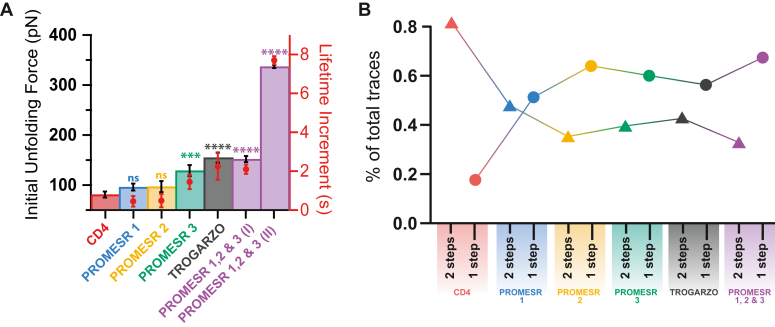
**Mechanical stability and unfolding of CD4D1D2 and PROMESR.***A*, comparison of initial unfolding forces for CD4D1D2 domains in the presence of each PROMESR, Trogarzo, and combination of all PROMESR. The difference of initial unfolding force between CD4 and CD4 in presence of PROMESR 3, Trogarzo, and PROMESR 1, 2, and 3 are statistically significant with a *p* value of 0.0004, 0.0001, and 0.0001, respectively. The *right axis* in *red* represents the mechanical unfolding lifetime increment of the tandem CD4D1D2 upon SURPOMERS binding. Force is reported as mean ± SEM. *B*, fraction of unfolding events for CD4D1 and D2 domains in one (*triangles*) or two steps (*circles*) for each combination of CD4D1D2 and PROMESR. CD, cluster of differentiation; PROMESR, protein mechanical stability regulator.

To determine the ability of the PROMESR molecules to alter the mechanics of CD4 domains, we performed the smAFS experiment in the presence of PROMESR in a ratio 1:5, protein:PROMESR. Starting with PROMESR 1, we observe the same step size but with a significant shift in the number of events for each one, with the peak corresponding to one step unfolding, at 20.6 ± 1 nm, as the more prominent one (Fig. 4*C*). We also observe a slight increment in the initial unfolding force with respect to CD4D1D2 alone, being this force 95 ± 7 pN (Fig. 4*C*). In the case of PROMESR 2, we measure a very similar step size with distribution with initial unfolding force of 97 ± 11 pN (Fig. 4*D*); and for PROMESR 3, similar step size but in this case the increment in initial unfolding force is quite significant at 129 ± 11 pN (Fig. 4*E*). However, the most substantial increment in force occurs with the combined action of the three PROMESR by which the initial unfolding force shows two populations, population I peaking at 152 ± 6 pN (Fig. 4*F*), which is similar to the mechanical effect of Trogarzo (16), and a population II with higher average unfolding force of 336 ± 3 pN, which represents an increment of over 4-fold with respect to CD4D1D2 alone. We believe that this is probably the result of different combinations of PROMESR in the mix. Initial unfolding forces for all three PROMESR are summarized in Figure 5*A*. Interestingly, if we consider the pulling speed in the force-damp experiments, we can estimate the mechanical unfolding lifetime increment of the tandem CD4D1D2 upon PROMESR binding. For such calculation, we take as zero reference value the unfolding of CD4D1D2 alone. The binding of PROMESR increases the lifetime of the folded domains, a fraction of a second for PROMESR 1 and 2, up to several seconds for PROMESR 1, 2, and 3 together (right axis in Fig. 5*A*).

A clear effect that we observed is that with the binding of every PROMESR, CD4D1D2 changes its unfolding pattern from two steps to just one. As shown in Figure 5*B*, in the case of CD4D1D2, about 80% of the traces show two-step unfolding and following a regular pattern in which D2 unfolds first. In the presence of PROMESR 1, about 50% of the traces show one-step unfolding of about 21 nm. This percentage increases to over 60% for PROMESR 2 and 3, which is even more than the observed effect for Trogarzo. Interestingly, the more drastic shift is observed when combining the three PROMESR molecules with a proportion of one *versus* two steps of about 65% to 35% (Fig. 5*B*). This alteration in the mechanical unfolding clearly proves the effect of the PROMESR molecules in the mechanical integrity of the tandem. By avoiding the two-step unfolding, the small molecules are stabilizing the structure, likely reinforcing the β-strand network that connects both domains. Considering that the three PROMESR molecules seem to bind with elevated Docking score in the region connecting D1 and D2, these results are somehow expected.

## Discussion

In the past decades, high-throughput screening techniques have become the gold standard approach to drug discovery not only in research but also in the pharmaceutical industry. Additionally, the implementation of computational methods for structure-based virtual screening and molecular docking has boosted the capability of screening methods (35, 36, 37, 38, 39). These methods mostly utilize protein structures where small molecules, peptides, or ligands are docked to high affinity serving as initial step for further design or even experimental testing of alterations in a particular molecular process. This procedure can be applied to chemical libraries of compounds, providing a protocol for rapid testing of many molecules, thus considered a HTVS. In the present study, we apply a HTVS approach to search for small molecules that serve as mechano-regulators of CD4 domains, named PROMESR, increasing the mechanical stability in addition to altering the mechanical unfolding pathway. The alteration of the unfolding behavior, that is, the unfolding of the tandem occurs as a single step instead of separated steps, proves by itself that the binding of the PROMESR occurs, allowing this test to be even more sensitive that binding experiments based on enthalpic contributions. To the best of our knowledge, this is the first protocol that combining computational screening techniques with protein mechanical studies provides small molecules that alter the mechanical properties of a protein. We thus demonstrate that a highly developed drug discovery approach can be repurposed for a new functionality, which is mechano-regulation.

The identified PROMESR molecules alter the mechanical properties of human CD4, making the tandem D1D2 of CD4 to behave as a single unit, which means that structural integrity of the protein is reinforced. This is demonstrated by the unfolding of the tandem as a single step. One of the PROMESR increases the mechanical strength of the tandem over 50%; however, the combined action of the three molecules renders a CD4D1D2 tandem with a highly increased mechanical stability, with numerous unfolding events reaching over 330 pN. We believe that such increment is the results of high affinity interaction, especially in the intermediate region holding both domains, where a long β-strand is shared between the two domains. In fact, the three PROMESR show docking poses in that region, as demonstrated in Figure 3. Interestingly, only two residues of the D1D2 tandem, Ser79 and Asn30, are common to the interaction of the three PROMESR, which suggests that the combined interaction may entail double or even triple binding with no competition. This could result in the large increment in mechanical stability that we observe.

Our results represent a proof of concept of the possibility of searching molecules that act as mechano-regulators. This is important because controlling the mechanical stability of proteins may have important implications. It is well known that numerous diseases are related to structural changes in proteins that may results from mechanical perturbations. These changes may be introduced by mutations and therefore are not easily corrigible. A good example could be mutations that cause mechanical alterations in cardiac proteins, generating hypertrophic cardiomyopathies (40). Similarly, other mutations relating mechanical stability of proteins and disease have been identified (41). However, other biological processes such as protein transport across membrane pathways or nuclear pores (14, 42, 43, 44), the mechanoactivation of ion channels (35, 44, 45) or cancer cell development (46, 47), are associated to protein mechanics. Therefore, having a protocol that employs the same approaches as drug discovery techniques, combined with mechanical studies of the protein of interest, allows us altering the protein mechanical stability, making this approach very interesting in many disciplines related to cell and molecular biology.

In our case, we have chosen human CD4 as potential target for protein mechanical stabilization. The intended idea was to find small molecules whose docking pose had a high docking score in regions that are mechanically important, such as the D1D2 interface, and therefore are candidate to stabilize the protein. From over 100,000 compounds, we found only three molecules with high enough affinity, but our search criteria were purposely strict and akin to conditions used for drug discovery. Thus, our intention of creating a pharmacology-based approach has been successful in terms of mechanical stabilization of CD4 domains. Whether our PROMESR molecules may have an inhibitory effect of HIV-1, such as the case of Trozargo, is an open question that is beyond the initial scope of this work; however, we hypothesize that these molecules may be a good starting point for developing potential small molecule that alter HIV-1 entry, as they may restrict the molecular interactions of CD4 and gp120 binding but also avoid conformational alterations in CD4. Also, in this initial analysis, only domains D1 and D2 have been studied. Including D3 and D4 might be of interest for further development of mechano-modulators for inhibiting HIV-1 entry. In fact, conformational changes in the interface between domains D2 and D3 has been suggested to play a role in HIV-1 binding (48).

Finally, it is important to mention that this approach could also be used to decrease the mechanical stability of proteins. Thus, targeting regions that serve as anchoring point of mechanical elements may create a binding competition that results in diminished stability. An example could be the β-strand complementation that occurs between protein modules of bacterial adhesin molecules such as microbial surface components recognizing adhesive matrix molecules or bacterial pili. This type of interaction between domains is extremely strong and the main responsible for the success of many bacterial infections (14, 49).

We conclude that the many possibilities of mechano-regulators as molecules that modify the mechanical stability of proteins in a controlled manner, opens new possibilities in experimental protein studies, as virtually any protein could be the subject of a search of such molecules. The approach is simple, it is well established, and mostly requires knowledge about the mechanical properties and structure of the protein under study. Hence, we demonstrate that protein mechanics brings new molecular interactions and functionalities for drug discovery approaches, not considered before, thus expanding the applicability of these techniques.

## Experimental procedures

### Rational identification of CD4 surface receptor mechanical regulators: compound selection criteria

A virtual screening protocol was set up to identify small molecules with the ability to modify the mechanical properties of CD4. In this sense, the key properties of an ideal CD4 mechano-modulator were established in this work as follows: (1) should display a strong (at least nanomolar) binding to CD4; (2) should not compete directly with MHCII or gp120 binding to CD4; (3) should have optimal ADMEt properties; and (4) should enjoy of a complete freedom of operation at the industrial property level. In addition, the commercial availability and price of the compounds identified were also considered.

### Receptor preparation

To quantify the binding affinity of known molecules to CD4, the following procedure was followed: The structure of residues 1 to 178 of the human T cell surface glycoprotein CD4 was downloaded from the protein data bank (PDB ID: 1WIP) and prepared with the Protein Preparation Wizard of Schrödinger suite. The preprocessing was carried out with default methods and H-bond refinement was carried out with default pH value 7. Three distinct Glide Grid files with an enclosing box of ca. Forty six angstroms were created using the above-mentioned structure, centered on Ser23, Leu95, and Val146, respectively, which properly cover CD4-D1, CD4-D2, and the CD4-D1D2 interface, respectively.

### Ligand preparation and docking

Molecules (ligands) to be screened were downloaded from the ZINC database, a free database of commercially available compounds for virtual screening. Approximately 100,000 compounds of the lead-like subset of the ZINC database were prepared for docking using LigPrep 5, with the OPLS_2005 force field. To set the ionization and tautomerization state of compounds at a pH range of 6 to 8, Epik v16207 was used, with a maximum number of four generated structures. The binding affinity of 100,000 lead-like prepared compounds was estimated through a three steps docking protocol summarized as follows: (a) a HTVS Glide procedure of all the compounds and a subsequent filtering-off of those that did not display a single pose with a binding affinity (docking score) above a predefined lower-limit value of −5 kcal/mol; (b) an standard precision level Glide docking procedure applied to those compounds overcoming the HTVS filter and a subsequent selection of those compounds displaying consistent binding affinities (docking scores) below −5 kcal/mol in all their poses for at least one site; and (c) an XP Glide docking and selection of top binders (10%).

### Forbidden binding sites

The compounds overcoming the mentioned energy barriers, we subjected to a second analysis focused on the identification of their binding regions, aimed at discarding drug candidates that would competitively interfere with either MHCII or gp120 binding. In this line, two distinct regions with “forbidden residues” were defined in the CD4 structure, a region defined by residues 35 to 52, 55 to 60, and 164 to 165 (MHCII-binding epitope) and a region around residues 29, 35, 43 to 47 and 59 in D1 tip (gp120-binding epitope). The first region, MHCII, is related to the natural function of CD4, therefore no interaction of PROMESR is desired. In the case of gp120, potential future experiments using viral elements would not interact with PROMESR molecules, thus focusing only on mechanical effects.

### Final selection criteria

The ADMEt properties of the noncompetitive and efficient CD4 binders identified so far were estimated using the Qikprop module of the Schrödinger (https://newsite.schrodinger.com/platform/products/qikprop/) software (Schrödinger Release 2017-4: Canvas, Schrödinger, LLC, NY, 2017). Also, the conclusions derived from a deep analysis of the patentability of these compounds, their market price, and availability were key to selecting the final molecules to be tested as novel CD4 mechano-modulators.

### Protein expression

Gene encoding (I91)2-CD4D1D2-(I91)2 chimeric polyprotein construct was designed and optimized for expression in *Escherichia coli* (Life Technologies). Here, two additional cysteine residues were added in the C terminus, which helps for sample immobilization on the gold surface. Standard DNA manipulation protocols were used to clone the construct into the pQE80L expression plasmid (Quiagen). C41 strand competent cells *E coli* (Novagen) were used for protein expression. Transformed competent cells were grown in 750 ml of LB media at 37 °C until an *A*600 of around 0.6 was reached. Then protein expression was introduced by 1 mM of IPTG and further incubation at room temperature for 4 h. Cells were then centrifuged, and a gentle cell lysis protocol was used to avoid damage to the expressed polyproteins. The sample was then purified first by HisTag affinity chromatography using a gravity column filled with HisPur Cobalt resin (Thermo Fisher Scientific) and second by size-exclusion chromatography using a Superdex 200 HR column (GE Healthcare). The final elution buffer was Hepes 10 mM pH 7, NaCL 150 mM, and EDTA 1 mM. The sample was further concentrated using ultrafiltration Amicon 3k filters (Millipore). The final protein concentration was estimated to be around 1 mg ml^−1^ using a Nanodrop (Thermo Fisher Scientific). Then, the samples were snap frozen in liquid nitrogen and stored at −80 °C.

### Single-molecule force spectroscopy

All single-molecule force spectroscopy force-ramp experiments were performed on an atomic force spectrometer AFS-1 (Luigs & Neumann). BioLever cantilevers from Olympus/Bruker were used with a spring constant of around 6 pN nm^−1^. The spring constant was measured before each experiment using the equipartition theorem within a software built-in procedure. Data was recorded between 0.5 to 4 kHz for the force-ramp measurements. For experiments, the force was ramped at 33pN·s^−1^ until 485 pN (starting from 10 pN pushing F < 0). This force value was held for 5 s to ensure the I91 subdomains unfolding. All atomic force microscopy experiments were carried out at room temperature (∼24 °C) in Hepes buffer at pH 7. Typically, 40 μl of the protein sample (∼5 μM concentration) was left around 20 min for adsorption on a fresh gold coated surface, using gold evaporation (Oerlikon UNIVX350). After the adsorption time, the sample was then rinsed of the gold surface by the Hepes buffer to remove unbounded protein sample just before starting the measurements. In these experiments, in presence of different PROMESR, these molecules were added to the Hepes buffer in a ratio 1:5 (protein:PROMESR).

### Cytotoxicity assay

Following 2 weeks of TZM lb passage, the cell cultures were prepared for cytotoxicity assay utilizing PROMESR. A colorimetric method employing 3-(4,5-dimethylthiazol-2-yl)-2,5-diphenyltetrazolium bromide (MTT) was employed to measure metabolic activity through a reduction occurring in the mitochondria of viable cells. In viable cells, this reaction caused a color change from yellow to purple, measured at 590 nm. For this purpose, the cell culture was cultivated in a P96-well plate. Once optimal cell coverage was achieved, the assay commenced. Each well received treatment with varying concentrations of PROMESR and Ibalizumab. After the designated incubation period, media from each well was removed, and a mixture of FBS-free media and MTT solution (in a 1:1 ratio) was added (100 ml) to each well. Incubation was conducted at 37 ºC for 3 h. Following incubation, 150 ml of MTT solvent was added to each well, followed by incubation, covering with foil, and shaking on an orbital shaker at room temperature for 15 min. Finally, the absorbance at 590 nm for each well was measured using Victor equipment.

## Data availability

Data supporting the findings of this study are available from the corresponding author upon reasonable request.

## Supporting information

This article contains supporting information.

## Conflict of interest

The authors declare that they have no conflicts of interest with the contents of this article.

## References

[1] Schonfelder J., Alonso-Caballero A., De Sancho D., Perez-Jimenez R. (2018). The life of proteins under mechanical force. Chem. Soc. Rev..

[2] Gupta R., Toptygin D., Kaiser C.M. (2020). The SecA motor generates mechanical force during protein translocation. Nat. Commun..

[3] Seifert C., Gräter F. (2013). Protein mechanics: how force regulates molecular function. Biochim. Biophys. Acta.

[4] Vogel V., Sheetz M. (2006). Local force and geometry sensing regulate cell functions. Nat. Rev. Mol. Cell Biol..

[5] Dufrene Y.F., Pelling A.E. (2013). Force nanoscopy of cell mechanics and cell adhesion. Nanoscale.

[6] Klotzsch E., Stiegler J., Ben-Ishay E., Gaus K. (2015). Do mechanical forces contribute to nanoscale membrane organisation in T cells?. Biochim. Biophys. Acta.

[7] Yusko E.C., Asbury C.L. (2014). Force is a signal that cells cannot ignore. Mol. Biol. Cell.

[8] Lim C.G., Jang J., Kim C. (2018). Cellular machinery for sensing mechanical force. BMB Rep..

[9] Pines M., Das R., Ellis S.J., Morin A., Czerniecki S., Yuan L. (2012). Mechanical force regulates integrin turnover in Drosophila *in vivo*. Nat. Cell Biol..

[10] del Rio A., Perez-Jimenez R., Liu R., Roca-Cusachs P., Fernandez J.M., Sheetz M.P. (2009). Stretching single talin rod molecules activates vinculin binding. Science.

[11] Pannekoek W.J., de Rooij J., Gloerich M. (2019). Force transduction by cadherin adhesions in morphogenesis. F1000Res.

[12] Bausch-Fluck D., Hofmann A., Bock T., Frei A.P., Cerciello F., Jacobs A. (2015). A mass spectrometric-derived cell surface protein atlas. PLoS One.

[13] Gordon V.D., Wang L. (2019). Bacterial mechanosensing: the force will be with you, always. J. Cell Sci..

[14] Alonso-Caballero A., Schonfelder J., Poly S., Corsetti F., De Sancho D., Artacho E. (2018). Mechanical architecture and folding of E. coli type 1 pilus domains. Nat. Commun..

[15] Alegre-Cebollada J., Badilla C.L., Fernandez J.M. (2010). Isopeptide bonds block the mechanical extension of pili in pathogenic Streptococcus pyogenes. J. Biol. Chem..

[16] Perez-Jimenez R., Alonso-Caballero A., Berkovich R., Franco D., Chen M.W., Richard P. (2014). Probing the effect of force on HIV-1 receptor CD4. ACS Nano.

[17] Wiegand T., Fratini M., Frey F., Yserentant K., Liu Y., Weber E. (2020). Forces during cellular uptake of viruses and nanoparticles at the ventral side. Nat. Commun..

[18] Mathelie-Guinlet M., Viela F., Pietrocola G., Speziale P., Alsteens D., Dufrene Y.F. (2020). Force-clamp spectroscopy identifies a catch bond mechanism in a Gram-positive pathogen. Nat. Commun..

[19] Spaulding C.N., Schreiber H.L.T., Zheng W., Dodson K.W., Hazen J.E., Conover M.S. (2018). Functional role of the type 1 pilus rod structure in mediating host-pathogen interactions. Elife.

[20] Rivas-Pardo J.A., Badilla C.L., Tapia-Rojo R., Alonso-Caballero A., Fernandez J.M. (2018). Molecular strategy for blocking isopeptide bond formation in nascent pilin proteins. Proc. Natl. Acad. Sci. U. S. A..

[21] Perez-Jimenez R., Garcia-Manyes S., Ainavarapu S.R., Fernandez J.M. (2006). Mechanical unfolding pathways of the enhanced yellow fluorescent protein revealed by single molecule force spectroscopy. J. Biol. Chem..

[22] Li H., Carrion-Vazquez M., Oberhauser A.F., Marszalek P.E., Fernandez J.M. (2000). Point mutations alter the mechanical stability of immunoglobulin modules. Nat. Struct. Biol..

[23] Zuo J., Zhan D., Xia J., Li H. (2021). Single-molecule force spectroscopy studies of missense titin mutations that are likely causing cardiomyopathy. Langmuir.

[24] Suay-Corredera C., Pricolo M.R., Velázquez-Carreras D., Pathak D., Nandwani N., Pimenta-Lopes C. (2021). Nanomechanical phenotypes in cardiac myosin-binding protein C mutants that cause hypertrophic cardiomyopathy. ACS Nano.

[25] Hu X., Li H. (2014). Force spectroscopy studies on protein–ligand interactions: a single protein mechanics perspective. FEBS Lett..

[26] Cerqueira N.M., Sousa S.F., Fernandes P.A., Ramos M.J. (2009). Virtual screening of compound libraries. Methods Mol. Biol..

[27] Irwin J.J., Shoichet B.K. (2016). Docking screens for novel ligands conferring new biology. J. Med. Chem..

[28] Lavecchia A., Di Giovanni C. (2013). Virtual screening strategies in drug discovery: a critical review. Curr. Med. Chem..

[29] Friesner R.A., Banks J.L., Murphy R.B., Halgren T.A., Klicic J.J., Mainz D.T. (2004). Glide: a new approach for rapid, accurate docking and scoring. 1. Method and assessment of docking accuracy. J. Med. Chem..

[30] Freeman M.M., Seaman M.S., Rits-Volloch S., Hong X., Kao C.Y., Ho D.D. (2010). Crystal structure of HIV-1 primary receptor CD4 in complex with a potent antiviral antibody. Structure.

[31] Kumar S., Sharma P.P., Shankar U., Kumar D., Joshi S.K., Pena L. (2020). Discovery of new hydroxyethylamine analogs against 3CLpro protein target of SARS-CoV-2: molecular docking, molecular dynamics simulation, and structure–activity relationship studies. J. Chem. Inf. Model..

[32] Friesner R.A., Murphy R.B., Repasky M.P., Frye L.L., Greenwood J.R., Halgren T.A. (2006). Extra precision glide: docking and scoring incorporating a model of hydrophobic enclosure for protein−ligand complexes. J. Med. Chem..

[33] Wang J.-H., Meijers R., Xiong Y., Liu J.-H., Sakihama T., Zhang R. (2001). Crystal structure of the human CD4 N-terminal two-domain fragment complexed to a class II MHC molecule. Proc. Natl. Acad. Sci. U. S. A..

[34] Lipinski C.A. (2004). Lead- and drug-like compounds: the rule-of-five revolution. Drug Discov. Today Tech..

[35] Gerhold K.A., Schwartz M.A. (2016). Ion channels in endothelial responses to fluid shear stress. Physiology.

[36] Gao W., Hasan H., Anderson D.E., Lee W. (2022). The role of mechanically-activated ion channels Piezo1, Piezo2, and TRPV4 in chondrocyte mechanotransduction and mechano-therapeutics for osteoarthritis. Front. Cell Dev. Biol..

[37] Sianati S., Kurumlian A., Bailey E., Poole K. (2019). Analysis of mechanically activated ion channels at the cell-substrate interface: combining pillar arrays and whole-cell patch-clamp. Front. Bioeng. Biotechnol..

[38] Jin Z., Du X., Xu Y., Deng Y., Liu M., Zhao Y. (2020). Structure of Mpro from SARS-CoV-2 and discovery of its inhibitors. Nature.

[39] Sadybekov A.A., Sadybekov A.V., Liu Y., Iliopoulos-Tsoutsouvas C., Huang X.-P., Pickett J. (2022). Synthon-based ligand discovery in virtual libraries of over 11 billion compounds. Nature.

[40] Poole K. (2022). The diverse physiological functions of mechanically activated ion channels in mammals. Annu. Rev. Physiol..

[41] Lewis A.H., Grandl J. (2021). Stretch and poke stimulation for characterizing mechanically activated ion channels. Methods Enzymol..

[42] Richardson J., Kotevski A., Poole K. (2021). From stretch to deflection: the importance of context in the activation of mammalian, mechanically activated ion channels. FEBS J..

[43] Stewart T.A., Hughes K., Stevenson A.J., Marino N., Ju A.L., Morehead M. (2021). Mammary mechanobiology - investigating roles for mechanically activated ion channels in lactation and involution. J. Cell Sci..

[44] Kefauver J.M., Ward A.B., Patapoutian A. (2020). Discoveries in structure and physiology of mechanically activated ion channels. Nature.

[45] Martinac B., Poole K. (2018). Mechanically activated ion channels. Int. J. Biochem. Cell Biol..

[46] Wirtz D., Konstantopoulos K., Searson P.C. (2011). The physics of cancer: the role of physical interactions and mechanical forces in metastasis. Nat. Rev. Cancer.

[47] Yankaskas C.L., Bera K., Stoletov K., Serra S.A., Carrillo-Garcia J., Tuntithavornwat S. (2021). The fluid shear stress sensor TRPM7 regulates tumor cell intravasation. Sci. Adv..

[48] Li W., Qin Z., Nand E., Grunst M.W., Grover J.R., Bess J.W. (2023). HIV-1 Env trimers asymmetrically engage CD4 receptors in membranes. Nature.

[49] Murthy S.E., Dubin A.E., Whitwam T., Jojoa-Cruz S., Cahalan S.M., Mousavi S.A.R. (2018). OSCA/TMEM63 are an evolutionarily conserved family of mechanically activated ion channels. Elife.

