# Supplementary material for: High-throughput virtual search of small molecules for controlling the mechanical stability of human CD4
Source: J Biol Chem. 2024 Mar 2;300(4):107133. doi: 10.1016/j.jbc.2024.107133 (PMC11065764; doi:10.1016/j.jbc.2024.107133)
Supplement: Supporting information [file mmc1.docx]

**Supplementary Information for**

**High-Throughput virtual search of small-molecules for controlling the mechanical stability of human CD4**

Antonio Reifs^1^, Alba Fernandez-Calvo^1^, Borja Alonso-Lerma^2^, Jörg Schönfelder^2^, David Franco^3^, Mariano Ortega-Muñoz^4^, Salvador Casares^5^, Concepcion Jimenez-Lopez^6^, Laura Saa^7^, Aitziber L. Cortajarena^7,8^, David De Sancho^9,10^, Eider San Sebastian^10, *^ & Raul Perez-Jimenez^1,8,*^

^1^Center for Cooperative Research in Biosciences (CIC bioGUNE), Basque Research and Technology Alliance (BRTA), Bizkaia Technological Park, Building 800
48160 Derio-Bizkaia (Spain).

^2^CIC nanoGUNE BRTA, Tolosa Avenue 73, San Sabestian 20018, Spain

^3^Glaxosmithkline, Rue de l'Institut 89, 1330 Rixensart, Belgium.

^4^Department of Organic Chemistry, Faculty of Science, University of Granada, 18071 Granada, Spain.

^5^Department of Physical Chemistry, Faculty of Science, University of Granada, 18071 Granada, Spain.

^6^Department of Microbiology, University of Granada,18002 Granada, Spain.

^7^Center for Cooperative Research in Biomaterials (CIC biomaGUNE), Basque Research and Technology Alliance (BRTA), Paseo de Miramón 194, Donostia-San Sebastián, 20014, Spain

^8^Ikerbasque, Basque Foundation for Science, Plaza Euskadi 5, 48009, Bilbao, Spain

^9^Donostia International Physics Center (DIPC), Manuel Lardizabal Ibilbidea 4, 20018 San Sebastian, Spain.

^10^Applied Chemistry Department, Faculty of Chemistry, University of the Basque Country (UPV/EHU), Manuel Lardizabal Ibilbidea 3, 20018 San Sebastian, Spain.

*Corresponding authors: R. Perez-Jimenez ([raulpjc@cicbiogune.es](mailto:raulpjc@cicbiogune.es)) and Eider San Sebastian ([eider.sansebastian@ehu.eus](mailto:eider.sansebastian@ehu.eus))

Validation of the docking protocol.

The crystal structure of any receptor deposited in the PDB needs a “preparation” process prior to be used in any virtual docking study. The “preparation” steps may include the addition of missing hydrogens, missing sidechains and/or loops, assigning bond orders, creating zero-order bond to metals, and optimization of H-bond networks, among others. Also, the ligands to be docked and the docking protocol itself, such as the conditions and parameters to be used, must be properly set. Even though virtual docking techniques have widely been used and proven valid, it is still interesting to validate the virtual structures and protocols used in each case. In this sense, the ideal validation would imply to computationally reproduce an experimentally co-crystallized receptor-ligand complex. Since no CD4-small molecule complex has ever been reported or crystallized previously, in this study, an alternative protein-small molecule complex structure was used, PDB ID= 8GCZ (https://doi.org/10.2210/pdb8gcz/pdb), where (3S,5S)-5-(aminomethyl)-N-(4-chloro-3-fluorophenyl)-1-(4-methylpiperazine-1-carbonyl)piperidine-3-carboxamide (ZXC-I-090 or YZI), an HIV-1 GP120 antagonist, is cocrystallized with HIV-1 LM/HT Clade A/E CRF01 GP120 Core.

**Supplementary Figure 1** shows the interactions stablished between receptor residues and functional groups in YZI ligand, as found experimentally in PDB 8GCZ (with no preparation or virtual docking). Two H-bonds are stablished between the amine and amide motifs of the ligand ZXC-I-090, and ASP368 and ASN425 residues, respectively, as well as pi-pi stacking interactions between the 4-chloro-3-fluorophenyl fragment of the ligand and residue TRP427. Also, the methylpiperazine fragment of the ligand stays essentially exposed to the solvent.

The pre-processing of the receptor was carried out with the Protein Preparation Wizard of Schrödinger suite, using default methods, and H-bond refinement was carried out with default pH value 7. A Glide Grid files with an enclosing box of ca. 30 Å was created using the above-mentioned structure, centred on the ligand ZXC-I-090 (pink solid-line box in **Supplementary Figure 2**) which properly covers not only the experimentally identified binding site of the ligand (black dashed square in **Supplementary Figure 3**), but other pockets found in the receptor.

The ligand (ZXC-I-090) was prepared for docking using LigPrep 5, with the OPLS_2005 force field. To set the ionization and tautomerization state of compounds at a pH range of 6–8, Epik v16207 was used, with a maximum number of 4 generated structures. Two alternative protonation states were found to be plausible for the 4-methylpiperazine fragment at the pH range studied, being the protonated state favoured over the unprotonated one, both in terms of free and complexed ligands. The binding mode of prepared ZXC-I-090 ligands to the prepared receptor grid was estimated through a High Throughput Virtual Screening (HTVS) Glide procedure, with standard settings. The computationally obtained receptor-ligand complexes were compared to the experimentally obtained one.

Importantly, the ligand interaction diagrams derived from the computationally docked ligand-receptor complexes **Supplementary Figure 3,** display the same exact interactions as the ones described experimentally (See **Supplementary Figure 2**) for comparison) Two H-bonds are stablished between the amine and amide motifs of the ligand ZXC-I-090 , and ASP368 and ASN425 residues, respectively, as well as pi-pi stacking interactions between the 4-chloro-3-fluorophenyl fragment of the ligand and residue TRP427. Also, the methylpiperazine fragment of the ligand stays essentially exposed to the solvent.

Supplementary Figure 1. Left, detail of the active site of the Clade A/E CRF01 GP120 Core in complex with ZXC-I-090 as found in the crystal structure; Right, ligand interaction diagram. In the diagram, residues are represented as coloured spheres, labelled with the residue name and residue number. The colours indicate the residue (or species) type: red—acidic (Asp, Glu); green—hydrophobic (Ala, Val, Ile, Leu, Tyr, Phe, Trp, Met, Cys, Pro); purple—basic (Hip, Lys, Arg); blue—polar (Ser, Thr, Gln, Asn, His, Hie, Hid); light gray—other (Gly, water). Interactions with the protein are marked with lines between ligand atoms and protein residues: Solid pink—H-bonds to the protein backbone; Dotted pink—H-bonds to protein side chains; Green—pi-pi stacking interactions; Orange—pi-cation interactions. Ligand atoms that are exposed to solvent are marked with grey spheres. The protein “pocket” is displayed with a line around the ligand, coloured with the colour of the nearest protein residue. The gap in the line shows the opening of the pocket.

**Supplementary Figure 2**. View of the outer box of the grid generated with the Glide grid generation module to properly map distinct binding pockets found on the receptor. Pink solid-line box, enclosing box used computationally to generate the receptor grid; black dashed square, experimentally identified binding site of the ligand.

Supplementary Figure 3. Ligand interaction diagrams of the computationally docked ligand. In the diagram, residues are represented as coloured spheres, labelled with the residue name and residue number. The colours indicate the residue (or species) type: red—acidic (Asp, Glu); green—hydrophobic (Ala, Val, Ile, Leu, Tyr, Phe, Trp, Met, Cys, Pro); purple—basic (Hip, Lys, Arg); blue—polar (Ser, Thr, Gln, Asn, His, Hie, Hid); light gray—other (Gly, water). Interactions with the protein are marked with lines between ligand atoms and protein residues: Solid pink—H-bonds to the protein backbone; Dotted pink—H-bonds to protein side chains; Green—pi-pi stacking interactions; Orange—pi-cation interactions. Ligand atoms that are exposed to solvent are marked with grey spheres. The protein “pocket” is displayed with a line around the ligand, coloured with the colour of the nearest protein residue. The gap in the line shows the opening of the pocket. Shown are the diagrams for the ZXC-I-090 ligand, both with the 4-methylpiperazine fragment protonated (left) and unprotonated (right).


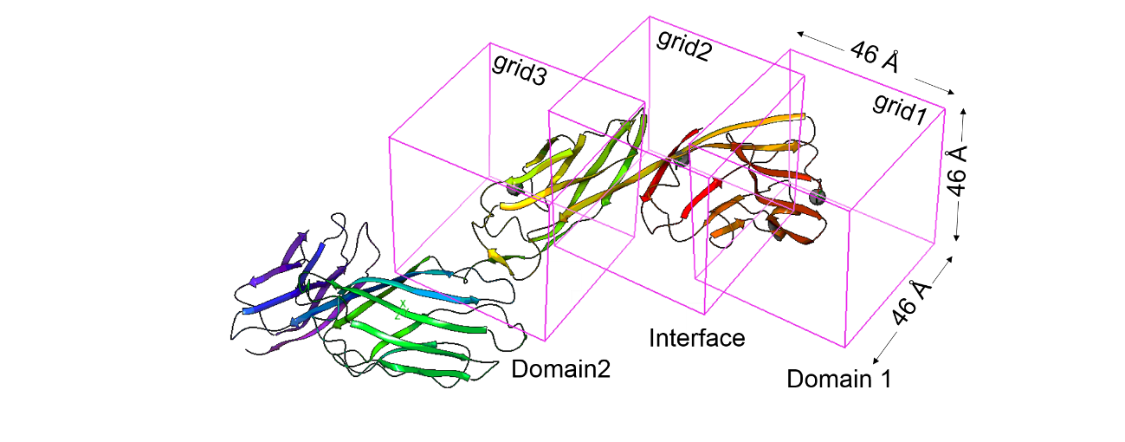


**Supplementary Figure 4.** View of the outer boxes of grids 1, 2 and 3 generated with the Glide grid generation module to properly map domains 1 and 2 of CD4 in 1WIP, as well as their interface. Grey spheres represent CA atoms of residues SER23, LEU95 and VAL146, where grids 1, 2 and 3 were centered, respectively.


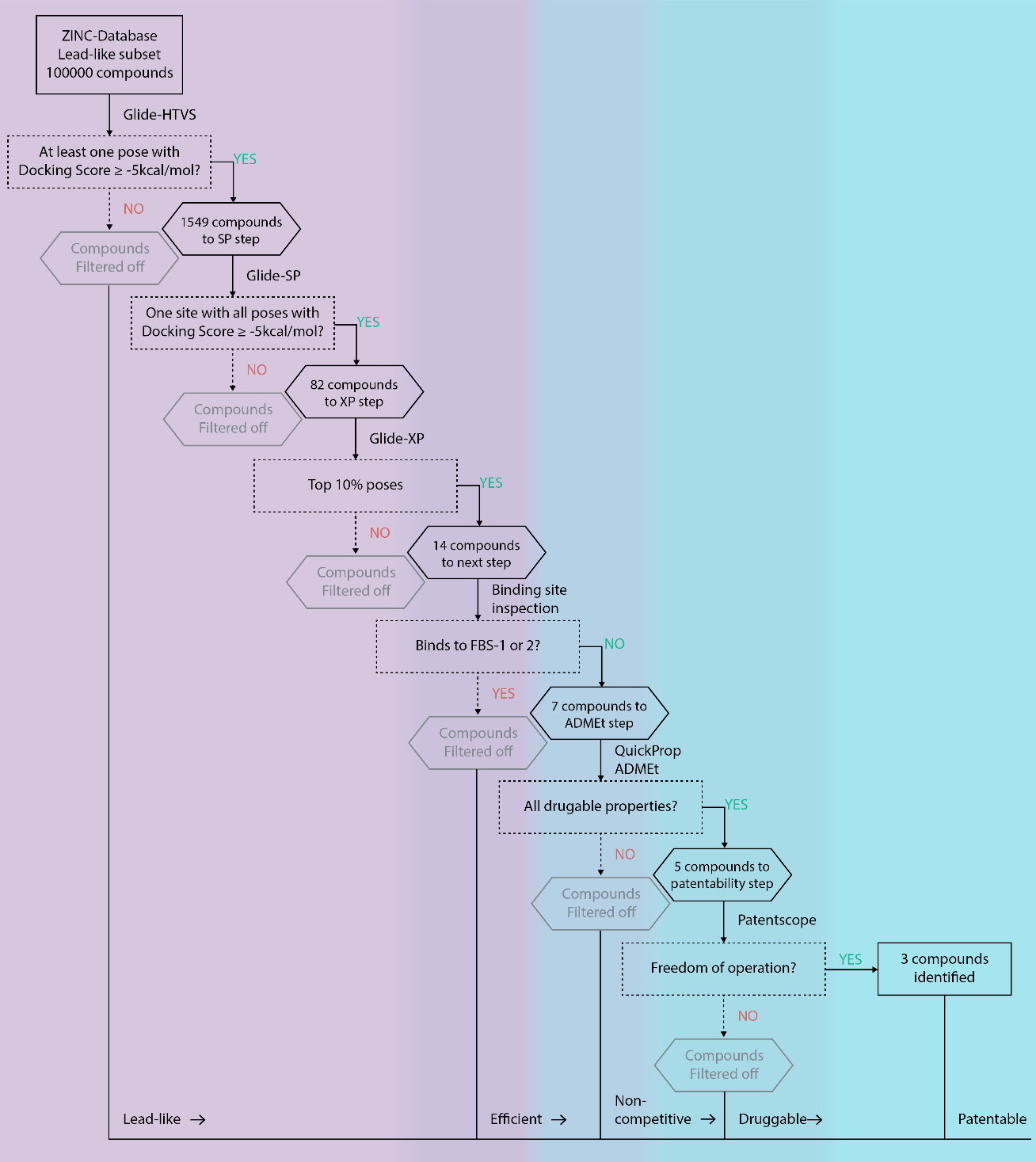


**Supplementary Figure 5.** Workflow used to identify CD4 mechano-modulators. The key properties of an ideal CD4 mechano-modulator were established as follows: (1) it should display a strong binding to CD4; (2) it should not compete directly with MHCII or gp120 binding to CD4; (3) it should have optimal ADMEt properties; and (3) it should enjoy a complete freedom of operation at the industrial property level.


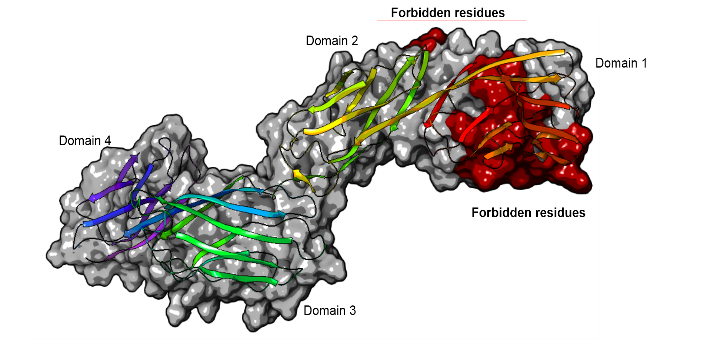


**Supplementary Figure 6. Forbidden binding sites.** CD4 (full length) surface and cartoon representation, where residues implicated in gp120 and/or MHCII binding (“Forbidden residues”) are indicated in red.


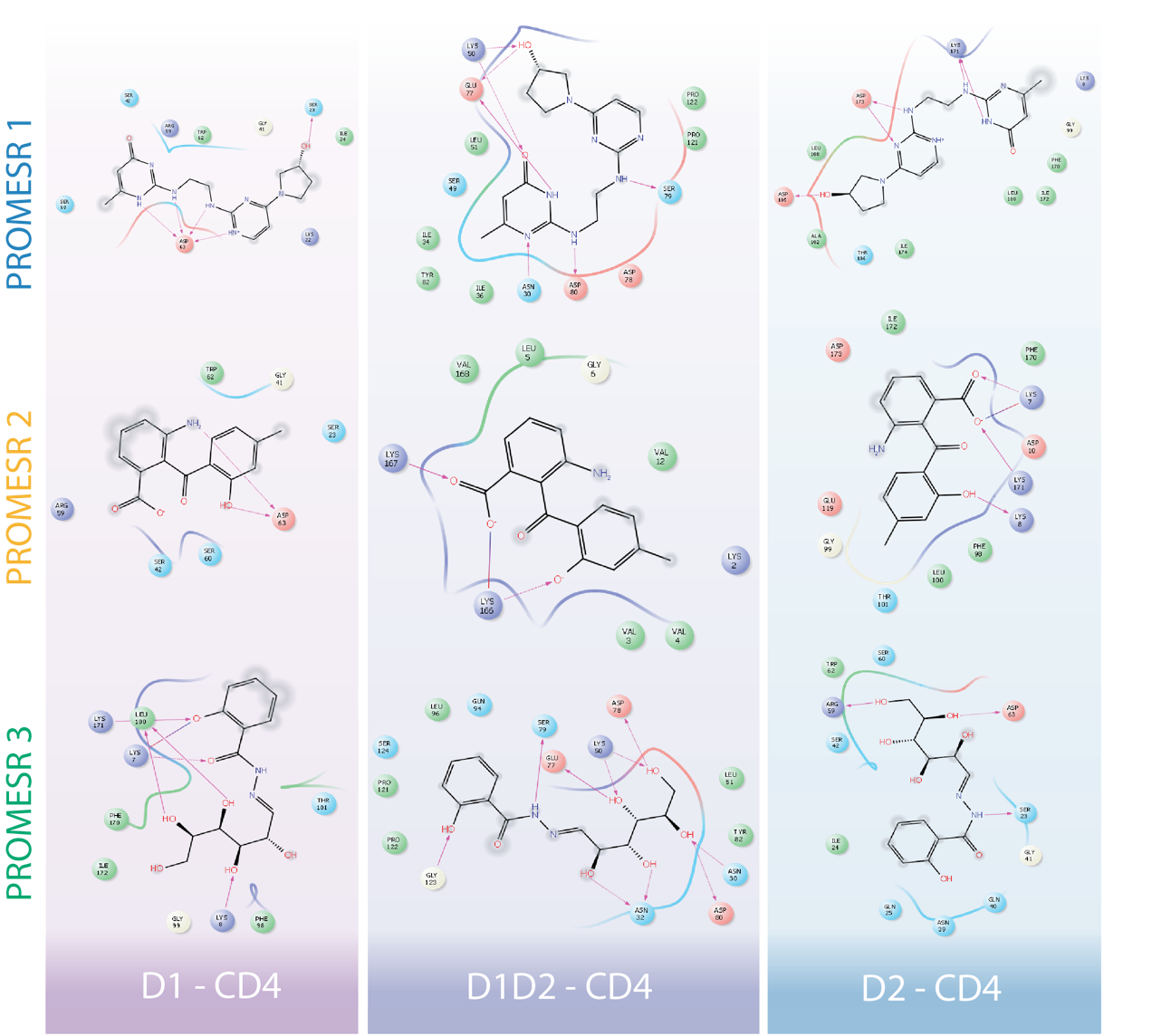


**Supplementary Figure 7.** Ligand interaction diagrams for best poses of PROMESR 1 (top), 2 (middle) and 3 (bottom) in domain 1 tip (left), domains 1 and 2 interface (middle) and domain 2 (right). CD4 residues are indicated as colored spheres. Color code: red: acidic (Asp, Glu), green: hydrophobic (Ala, Val, Ile, Leu, Tyr, Phe, Trp, Met, Cys, Pro), purple: basic (Hip, Lys, Arg), blue: polar (Ser, Thr, Gln, Asn, His, Hie, Hid), light gray: other (Gly, water). Interactions with the protein are marked with lines between ligand atoms and protein residues as follows: Solid pink: H-bonds to the protein backbone; Dotted pink: H-bonds to protein side chains, Green: pi-pi stacking interactions.


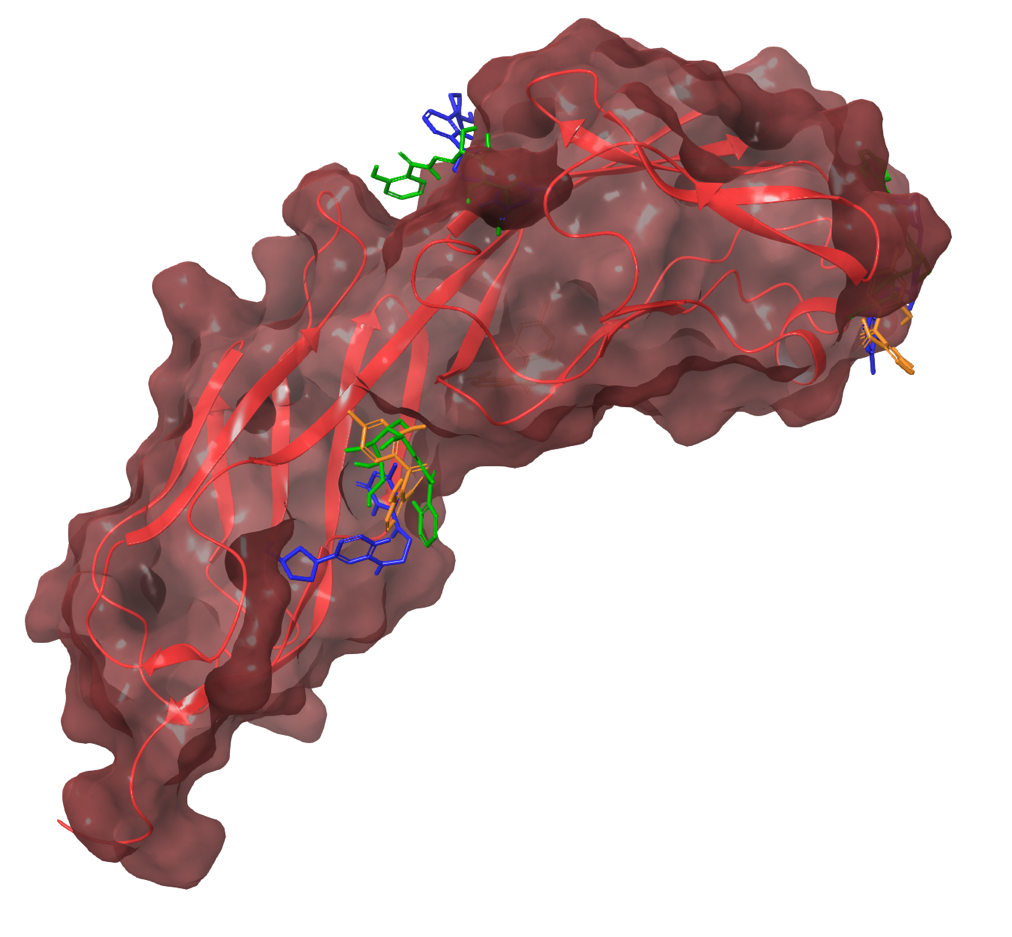


**Supplementary Figure 8.** Representation of CD4D1D2 structure with the three PROMESR simultaneously bound to CD4 in their best pose. PROMESR 1 (blue), PROMESR 2 (orange), and PROMESR 3 (green). The three locations are the same for all of them.


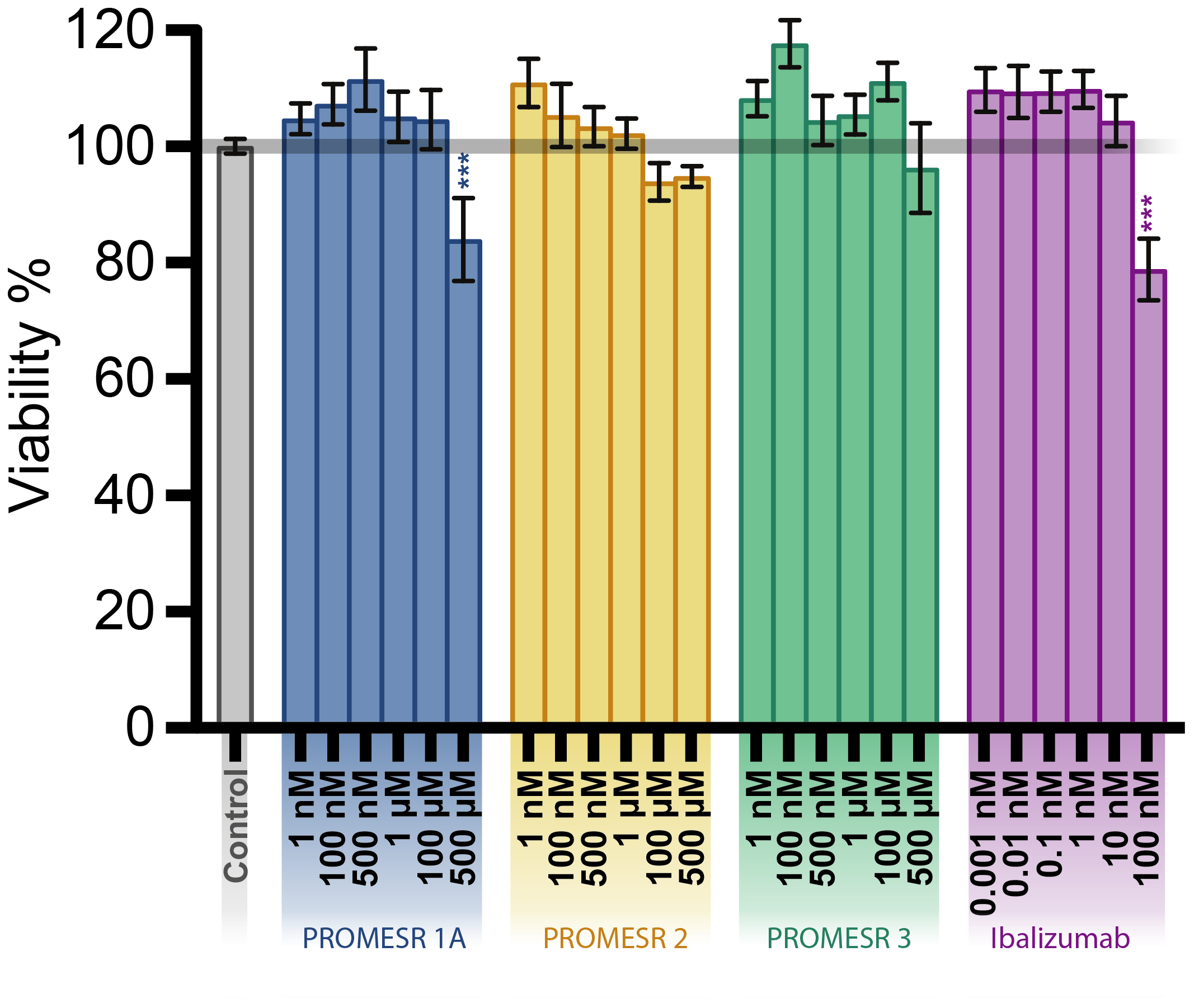


**Supplementary Figure 9.** Cytotoxicity assay using HEK 293 cells in absence (grey) as a control and presence of increasing concentrations of PROMESR and Trogarzo. The difference of viability percentage between control and cells in presence of 500 µM of Suremel 1A and 100 nM of Trogarzo are extremely statistically.


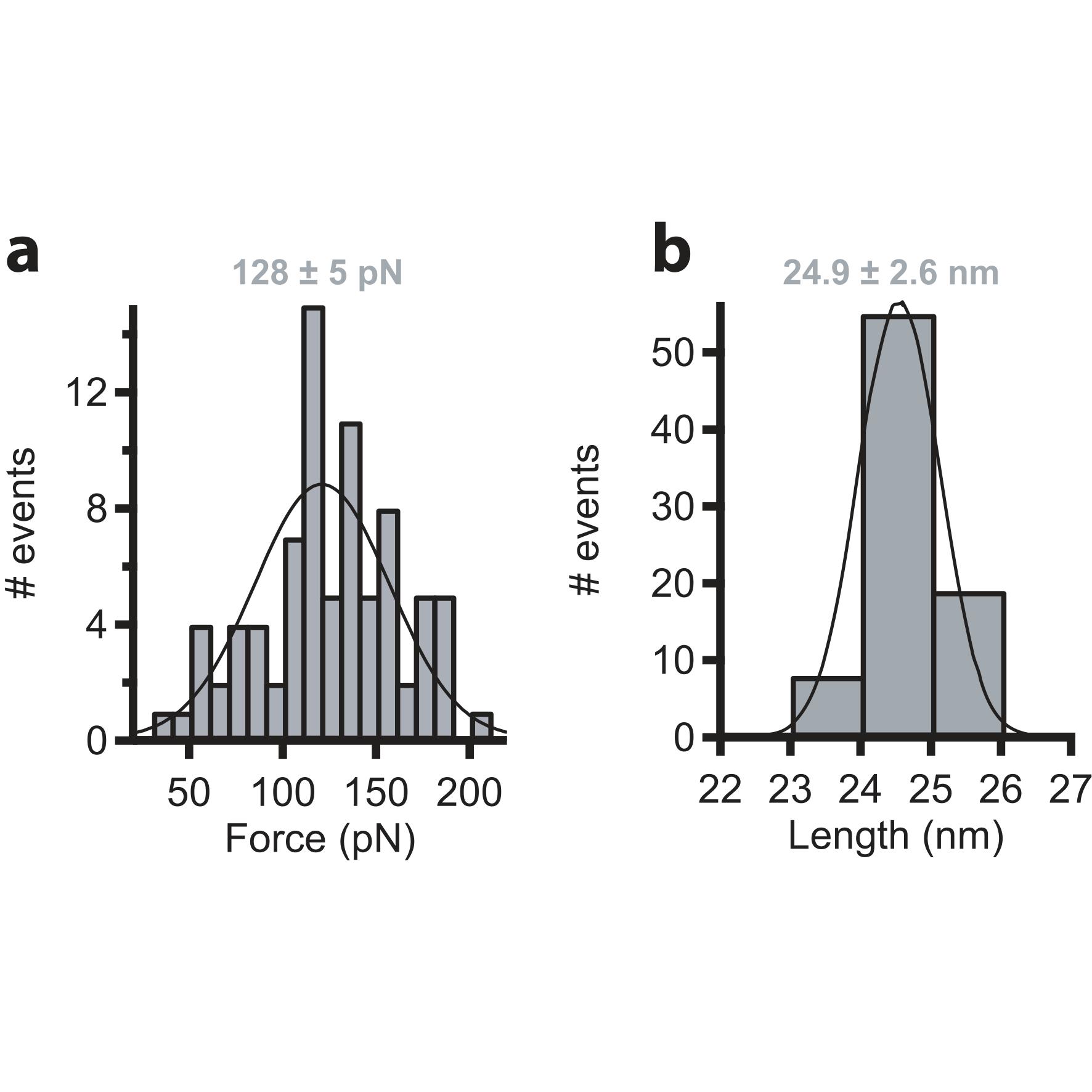


**Supplementary Figure 10. a)** Histogram of initial unfolding force of I91 (n = 82). We measured an unfolding force of 128 ± 4.5 pN. **b)** Histogram of step size for the unfolding of I91(n = 82). The unfolding of I91 Domain was measured at 24.9 ± 2.6 nm.

Property PROMESR 1 PROMESR 2 PROMESR 3

**Supplementary Table 1.** Physically significant descriptors and pharmaceutically relevant properties (ADME) of PROMESR 1, 2 and 3 were predicted with QikProp (**Schrödinger Release 2017-4:** QikProp, Schrödinger, LLC, New York, NY). A complete list of property description can be found at Schrödinger Release 2017–4: Canvas, Schrödinger, LLC, New York, NY, 2017.
